# A Report on the Ke Ola O Ka ‘Āina: ‘Āina Connectedness Scale

**DOI:** 10.3390/ijerph20043302

**Published:** 2023-02-13

**Authors:** Mapuana C. K. Antonio, Samantha Keaulana, LeShay Keli‘iholokai, Kaitlynn Felipe, Jetney Kahaulahilahi Vegas, Waimānalo Pono Research Hui, Waimānalo Limu Hui, Ke Ola O Ka ‘Āina Research Team and Thought Partners, Ilima Ho-Lastimosa

**Affiliations:** 1 Native Hawaiian and Indigenous Health, Office of Public Health Studies, University of Hawai‘i at Mānoa, Honolulu, HI 96822, USA; 2 Office of Public Health Studies, Thompson School of Social Work and Public Health, University of Hawai‘i at Mānoa, Honolulu, HI 96822, USA; 3 Ke Kula Nui O Waimānalo, Waimānalo, HI 96795, USA; 4 Department of Social Work, Thompson School of Social Work and Public Health, University of Hawai‘i at Mānoa, Honolulu, HI 96822, USA; 5 College of Tropical Agriculture and Human Resources, University of Hawai‘i at Mānoa, Honolulu, HI 96822, USA

**Keywords:** native Hawaiian, Indigenous, health, land, community-based, qualitative, scale development

## Abstract

Optimal health from a Native Hawaiian worldview is achieved by being pono (righteous) and maintaining lōkahi (balance) with all our relations, including our relationships as Kānaka (humankind) with ‘Āina (land, nature, environment, that which feeds) and Akua (spiritual realm). The purpose of this study is to explore the role of ‘Āina connectedness in Native Hawaiian health and resilience to inform the development of the ‘Āina Connectedness Scale. Qualitative methods were conducted with 40 Native Hawaiian adults throughout Hawai‘i. Three themes emerged: (1) ‘Āina is everything; (2) Connection to ‘Āina is imperative to health; and (3) Intergenerational health, healing, and resilience are reflected through intergenerational connectedness with ‘Āina. Qualitative findings, supplemented with a scoping review of land, nature, and cultural connectedness scales, led to the development of the ‘Āina Connectedness Scale, which examined the degree to which people feel connected to ‘Āina, with implications for future research. ‘Āina connectedness may address concerns related to health disparities that stem from colonization, historical trauma, and environmental changes and better our understanding of Native Hawaiian health by fostering stronger ties to land. Resilience- and ‘Āina-based approaches are critically important to health equity and interventions that aim to improve Native Hawaiian health.

## 1. Introduction

Health from a Kānaka Maoli or Kānaka ‘Ōiwi (Native Hawaiian) worldview emphasize the importance of being pono (morally upright, righteous) and maintaining a harmonious balance physically, mentally, spiritually, and emotionally. From this worldview, health includes interconnected relationships between Kānaka (humankind) and ‘Āina (land, nature, environment, that which feeds), which is reflected by the worldview of Lōkahi [1,2], demonstrating the idea that health is holistic and encompasses a spiritual and familial connection with land. Thus, connecting with oneself, others, and land is a mechanism of resilience that may foster health. This research focuses on Native Hawaiian health and resilience and applies a holistic and strengths-based approach that focuses on bettering the health of Native Hawaiians from a Native Hawaiian worldview. The purpose of this paper is to describe the process of developing and validating the ‘Āina Connectedness Scale to promote health equity research, specifically among Kānaka Maoli.

Kānaka Maoli viewed health as a sacred living force and required living in balance with their natural resources and surrounding environment. For instance, renowned Native Hawaiian leader, Dr. Noa Emmett Aluli, described the reciprocal relationship and interconnections between people and land through his philosophy “The health of the land is the health of the people”. This philosophy also reinforced the Native Hawaiian way of life, where people would deeply love and care for the land in exchange for the land nourishing them. Hawaiian proverbs and sayings, such as “He ali‘i ka ‘āina; he kauwā ke kanaka. The land is a chief; man is its servant” [‘Ōlelo No‘eau #531] further validate these worldviews.

Disruptions to this balanced lifestyle resulted from social and cultural determinants of health, including colonization, historical trauma, and changes in values and lifestyles, such as changes in connections to land and living sustainably from ‘Āina [3,4], which are inextricably linked to the health inequities [5] experienced today. Upon the arrival of James Cook in 1778, a decimation in population led to a drastic decline in the Native Hawaiian population by more than 90% [6]. Environmental disruptions resulting from changes in land tenureship and food systems were further exacerbated by significant events including privatization of land, a concept that was foreign to the Kānaka Maoli way of life; changes in land tenure from a collectivistic lifestyle and economy to one of capitalism; and rapid transitions in lifestyle which threatened the Kānaka Maoli way of life, including the illegal overthrow of the Hawaiian Kingdom and, thus, the illegal annexation, territorialization, and statehood of Hawai‘i [7,8]. Present-day forces continue to threaten the sacred life force of the land as demonstrated by Hawai‘i’s militarism and tourism-driven economy, with implications for environmental changes and adverse consequences on our water and life sources [9]. These socio-cultural determinants of health continue to be reflected in the health inequities experienced by Native Hawaiians [4,10]. The urgent need to respond to environmental determinants of health are not limited to Hawai‘i, and, in fact, climate change and environmental deterioration remain a prioritized topic for addressing determinants of health. Additionally, despite these threats and ecological determinants of health, other factors of resilience—such as the revitalization and resurgence of practices that allow for the reclaiming of Native Hawaiian identities and practices—reconnect Native Hawaiians with ‘Āina and Hawaiian ways of knowing, thereby fostering wellbeing [11,12,13,14,15,16,17].

This narrative of survivance, and thus, resilience, resonates with many other Indigenous communities across the globe [18,19]. For instance, Indigenous Peoples on average experience increased mortality with lower life expectancy compared with non-Indigenous counterparts, a health inequity that stems from the long-standing impacts of colonization, cultural and historical trauma, past and present mistreatments, and assimilative strategies [20,21]. These forms of oppression, supplemented with unethical research, have led to mistrust among western paradigms with research being connotated as one of the “dirtiest words” in Indigenous communities [22]. Nonetheless, Indigenous Peoples continue to thrive with a narrative of strength, survivance, and endurance, and are byproducts of generations and ancestors who endured colonialism and systems of oppression that continue to be perpetuated today [18,19].

Although these narratives of strengths, survivance, and endurance exist, the literature minimally portrays the resiliency of Native Hawaiians and Indigenous Peoples from this worldview. Furthermore, to date, we have little understanding of what supports resilience and how it promotes health and wellbeing, protecting Native Hawaiians from adversities. Understanding a person’s traits and external resources that aid in the ability to overcome adversity may shed light on resiliency as a mechanism of promoting health. While measures of resilience exist, most of these scales measure resilience on the individual level and focus on an individual’s ability to recover from a stressful event [23,24]. Recent research expands on this definition to include resilience at multiple levels; yet, few scales assess across levels. For instance, the American Psychological Association has proposed the Resilience Tool Kit [25] which applies the socio-ecological model framework [26] and considers a multi-dimensional approach to foster resilience. This framework helped to inform the development of a new and inclusive measure of resilience that considered strengths on multiple levels including individual characteristics and external resources [27].

In the Hawaiian Homestead Community Health Survey, health and health-related behaviors among Native Hawaiians were assessed using a comprehensive health survey developed and administered to adults residing on select Hawaiian Homestead Lands [28,29]. Preliminary findings from this research suggest that resilience consists of multiple factors [27]. While the measure of resilience demonstrated good model fit, Indigenous constructs of resilience—such as land connectedness, as proposed by Native Hawaiian participants in a qualitative study exploring resilience [2,30]—need to be considered and explored as strengths that may facilitate Native Hawaiian health and resilience.

While efforts to revitalize cultural practices flourish, land dispossession remains problematic, with implications for ‘Āina connectedness [30]. ‘Āina connectedness also aligns with goals set forth by the United Nations and health research that highlight the importance of exploring the relationship between the wellbeing of Indigenous people and their relationship with their land [31,32]. In fact, a report from the United Nations Conference on Sustainable Development explicitly cites the recognition of protecting planet Earth and the rights of nature as necessary to achieve balance and meet the needs of present and future generations [31]. National and global studies exploring nature, land, and environmental connectedness have been shown to be associated with favorable health outcomes [32]. According to a scoping review of land connectedness, 38 global scales exist and measure four major categories, including: (1) nature connectedness and relatedness scales, (2) attitudinal and values-based scales, (3) culturally and spiritually based scales, and (4) paradigm-based scales.

While a wealth of knowledge exists related to land connectedness, an integration of these scales will help to address current gaps in the literature, such as an exploration of ‘Āina connectedness from a Native Hawaiian worldview. For instance, culturally based scales were primarily developed and implemented with Indigenous communities to assess constructs such as intergenerational/ancestral knowledge, values, and ways of knowing that honor a deep relationship with nature and land. As an example, the Hawaiian Cultural Scale measured the degree to which Native Hawaiian adolescents know of, believe in, value, and practice elements of traditional Hawaiian culture which extends to include aspects of connection with ‘Āina [33]. The Awareness of Connectedness Scale (ACS) measured the degree to which Alaska Native youth endorsed the concept of interrelatedness between the self, family, community, and natural environment [34]. The Cultural Connectedness Scale (CCS) measured subscales of cultural connectedness, including self-efficacy, sense of self (present and future), school connectedness, and life satisfaction as a resiliency mechanism for mental health among First Nations, Métis, and Inuit youth [35,36]. An ‘Āina connectedness scale that measures these constructs specifically in relation to land, nature, and the environment may help to fill these gaps.

This study is the first step in addressing these gaps by integrating frameworks of land connectedness with Native Hawaiian perspectives of health and resilience. This study is also a response to community priorities of wanting to further explore constructs of ‘Āina connectedness as a factor of health.

## 2. Materials and Methods

This study comprised two major phases to develop the ‘Āina Connectedness Scale for health research in Native Hawaiian communities and utilized community-based participatory research (CBPR) approaches. Permission and support of this research was provided by the Waimānalo Pono Research Hui (WPRH) [37,38,39] and other Native Hawaiian communities. Ke Ola O Ka ‘Āina (KOOKA) Research Team and Thought Partners developed throughout the duration of the project and included communities and ‘Āina who contributed deeply to this project. The KOOKA Research Team and Thought Partners include partners across the Pae ‘Āina of Hawaii including the Ho‘okena Community, Hawai‘i Island, Kamāwaelualani, Kaua‘i Island, Maui Island, Moloka‘i Island, Lāna‘i Island, Waimānalo Community, and O‘ahu Island.

The name-giving process in the Native Hawaiian culture is a significant process that ultimately gives life to the essence of the named entity, in this case the naming of the project. For this process, the team consulted Ms. Jetney Kahaulahilahi Vegas, MPH, who explored the purpose and characteristics of the overall project. The project itself was viewed as having its own identity, thereby contributing to the name Ke Ola O Ka ‘Āina, which loosely translates to the life of the land. Through this name, we honor the various ‘Āina that continue to nourish us, as people, to engage in this work as people of the land.

### 2.1. Phase I: Qualitative Methods

#### 2.1.1. Participants

Key informant interviews and small focus groups were conducted with 40 Native Hawaiian community members and leaders engaged in aloha ‘Āina (a value and way of life that demonstrates a person’s deep love for and connection with the land) throughout all major islands of Hawai‘i. Interviewees were recruited through purposive and convenience sampling to ensure diversity in characteristics among participants and to ensure a relationship had been in place between the research team, comprised of Kānaka ‘Ōiwi scholars, and interviewees.

#### 2.1.2. Measures and Semi-Structured Interview Guide

All interviewees completed a demographic survey to ensure diversity among participants. Interviews and focus groups used a “talk story” methodology, which followed a semi-structured interview guide created based on the existing literature and consultation with community leaders and experts in the field. The semi-structured interview guide consisted of the following flow: oral re-consent; introductions; questions related to ‘Āina, health, and ‘Āina connectedness in relation to health; and closing remarks. Using thematic and grounded theory approaches, key themes of ‘Āina connectedness in relation to health were identified, which informed the development of the ‘Āina Connectedness Scale.

#### 2.1.3. Procedures and Data Analysis

To ensure community-based participatory research approaches were employed at every step of the research process, the Ke Ola O Ka ‘Āina research team sought permission to partner with the Waimānalo Pono Research Hui to conduct this research study in Waimānalo and with affiliated partners. In 2018, the Waimānalo Pono Research Hui developed the Pono Research Protocols and Rules of Engagement, which outline principles of engaging in research to ensure ethical and pono research in the Waimānalo community [37,38,39]. After receiving permission from the Waimānalo Pono Research Hui, the KOOKA research team obtained proper permission to proceed with the research process with, for, and by other Native Hawaiian communities. As part of the permission process, it was determined that all stories shared during the research process were owned by the various communities, organizations, and individuals who engaged in the qualitative research process. Additionally, the resulting ‘Āina Connectedness Scale would be owned by the Ke Ola O Ka ‘Āina Research Team and Thought Partners. This study was also approved by the university IRB.

Community experts and key members of the KOOKA research team provided support around recruitment and ensured the community was involved in all stages of the research process. Community experts helped to co-develop qualitative interview questions, co-analyze data and major themes, and provide support in dissemination. Key informant interviews comprised one interviewee, while small focus groups consisted of two to three interviewees. Interviews and focus groups were conducted by the first and second author with guidance provided by the community mentor of this project (last author). The interviews and focus groups ranged from 30 to 120 min and generally took place before, during, or after a community organized event (pre-COVID-19). Following strict mandates as a result of the Coronavirus Disease 2019 (COVID-19) pandemic, approximately 10 interviews took place via Zoom to ensure the safety of participants. All interviewees consented to participate in this study. The majority of interviewees consented to be audio recorded, which allowed for transcription of interviews verbatim. Two interviewees consented to participate but had a preference toward not being audio recorded, and therefore, two interviewers were present during the key informant interview to ensure quotes were captured verbatim.

All interviewees were thanked for their time and received a USD 25 gift card. Audio recorded interviews were transcribed and analyzed using a thematic and grounded theory approach [40]. The two lead authors of this study immersed themselves in all of the audio and transcriptions of each interview and focus group. Next, the three lead authors reviewed one of the focus groups collectively, to allow for the creation of a codebook with guidance provided by the community mentor of this project (last author). The remaining interviews and focus group were then reviewed independently by the two lead authors and later reviewed by the group. In the case of disagreement, the two lead authors consulted the third and last author and determined coding based on group consensus. In alignment with Kānaka ‘Ōiwi ways of knowing, the research team acknowledged that many of the themes are interconnected with one another. Therefore, the most salient and recurring codes were classified as a theme.

### 2.2. Phase II: Development of the ‘Āina Connectedness Scale and KOOKA Comprehensive Health Survey

The qualitative methods were further explored and reviewed with other existing measures of nature, land, environmental, and cultural connectedness, which led to the development of the ‘Āina Connectedness Scale. Questions were developed in collaboration with the KOOKA Research Team and Thought Partners to assess various domains of ‘Āina connectedness. The Ke Ola O Ka ‘Āina: ‘Āina Connectedness Scale was then included in a comprehensive health survey based on associated variables identified during qualitative interviews and focus groups. 

The comprehensive health survey was then cognitively tested with 20 Native Hawaiian adults throughout the major islands of Hawai‘i and with a primary focus on the items included in the newly developed ‘Āina Connectedness Scale. Cognitive interviews helped to establish integrity and preliminary validity for the ‘Āina Connectedness Scale and the comprehensive health survey. Cognitive interviews were conducted by the two lead authors of this study. Individuals who participated in the cognitive interviews were thanked for their time and received a USD 50 gift card for their feedback on our ‘Āina Connectedness Scale and KOOKA Comprehensive Health Survey. Questions were revised and updated based on feedback provided by the cognitive interviews and to ensure meaningfulness of each item.

## 3. Results

### 3.1. Phase I: Qualitative Methods

#### 3.1.1. Participant Characteristics

A total of 40 Native Hawaiian adults participated in qualitative interviews or small focus groups to share their knowledge, wisdom, stories, and lived experiences related to ‘Āina. Table 1 provides a breakdown in participant characteristics. Half of participants identified as being a kāne or male and the other half identified as being wāhine or female. About 1/3 of participants were mākua (or of a parent’s generational age when interviewed) and 2/3 were kūpuna (elders, or of a grandparent’s generational age when interviewed). A little less than half were from the island of O‘ahu, which tends to have more urban and remote communities, while the other half were from neighboring islands, which tend to be more rural or remote.

#### 3.1.2. Summary of Themes

Qualitative methods led to the development of three major themes: (1) ‘Āina is everything; (2) Connection to ‘Āina is imperative to Native Hawaiian health; and (3) Intergenerational health and resilience of people and communities are reflected through intergenerational connectedness with ‘Āina. A summary of themes with a codebook definition and example quotes are provided in Table 2.

### 3.2. Theme I: ‘Āina Is Everything

The most salient theme that resulted from the qualitative interviews and focus groups was the idea that “‘Āina is everything” (multiple interviewees, all islands). As described by one of the interviewees and a Native Hawaiian leader in their community, “‘Āina is everything. And is anything that nourishes us. ‘Āina is the land-from the mountains, to the sea, to us as people. It’s all connected…so when we connect with ‘Āina, we connect to each other”. Deeply embedded within this worldview is the notion that ‘Āina is everything, and therefore, we as people are ‘Āina. Some expressed such a deep love for and connection with ‘Āina that they described themselves as being part of ‘Āina. For instance, an interviewee from Hawai‘i Island explained this relationship as “I am the ‘āina”.

The reciprocal notion between ‘Āina and people are further validated by existing frameworks of health, such as the lōkahi triangle, which emphasize the importance of maintaining balance and harmony as people with ecological surroundings. This concept of lōkahi was often described by interviewees. Thus, what we put into ‘Āina will ultimately return back to us as people, demonstrating a strong need to maintain balance and harmony. To demonstrate this point, one of the interviewees described ‘Āina as “that which feeds. But I think of ‘Āina as not only land but the interconnectedness of all things… we are, we as humans, derive from the ‘Āina”. The concept “‘Āina” was also translated as the land and the environment by interviewees; however, most notably, interviewees also acknowledged the nourishment—physically, mentally, spiritually, and emotionally—that we receive from ‘Āina.

### 3.3. Theme II: Connection to ‘Āina Is Imperative to Native Hawaiian Health

Similar to Theme I, each participant described the significance of ‘Āina connectedness in relation to Native Hawaiian health. The ways in which people described this connection varied and included concepts related to mo‘okū‘auhau (loosely translated as genealogy in this context); respect for land and everything that nourishes people; kuelana, or a deep responsibility, birthright, or privilege that held people accountable to connect with ‘Āina; and through a connection to ‘ohana (family), community, and the lāhui (nation of Hawai‘i).

A connection to ‘Āina through genealogy was described in multiple ways. One of the most frequent forms of ‘Āina connectedness occurred through participants identifying ‘Āina as a family member, and thus, with their genealogical ties dating back to ‘Āina. Despite colonial attempts to erase ties with direct family members, many participants discussed their known genealogical ‘Āina descendance. Some cited this information as common knowledge, while others cited their known genealogical connection through the Kumulipo [41] and other mo‘olelo (loosely translated as stories) describing our relationship to ‘Āina, including the mo‘olelo of Hāloanakalaukapalili, a child of Wākea and Ho‘ohokukalani whose stillbirth resulted in the growth of a kalo (taro) plant that was cared for by his younger sibling, Hāloa.

Participants identified values such as respect and kuleana as agents of ‘Āina connectedness and as mechanisms of health, not just for the present generations but also to honor the generations of past and to leave sustainable ‘Āina for the generations to come. For instance, one of the interviewees indicated “We need to respect ‘Āina. And it’s shown in everything we do. We need to respect ‘Āina as our caregiver, as our provider, but also because our keiki are watching and learning as we mālama ‘Āina”. As showcased through this quote, respect was innately connected with a kuleana (birthright, privilege, and responsibility) to perpetuate aloha ‘Āina, our deep love for ‘Āina, with the understanding that we are ultimately the beneficiaries of our ancestors who cared for ‘Āina and must do the same for our present and future generations.

Connection to ‘Āina was identified as being imperative to health due to the connection that is fostered with and for each other, our ‘ohana (family), communities, and lāhui (nation of Hawai‘i). In alignment with the idea that ‘Āina is everything, including us as people, is the deeper understanding that we can foster social and cultural connectedness by connecting with ‘Āina and with one another. When we foster our relationships with others, we learn more about ‘Āina and the collective ways in which we connect with the land. By the same token, when we connect with ‘Aina, we have a deeper appreciation for nourishment that ‘Āina has provided to us as people for generations. As described by one of the participants, “‘Āina holds the story of people just as people honor the stories and practices to uphold ‘Āina”.

### 3.4. Theme III: Intergenerational Health, Healing, and Resilience of People and Communities Are Reflected through Intergenerational Connectedness with ‘Āina

The last theme builds on the previous two themes while emphasizing the importance of health and resilience of people and communities as reflected through intergenerational connectedness. This theme emphasizes the relational connection with ‘Āina across time, through intergenerational knowledge, and across levels, through the emphasis of individualistic and collectivistic health and resilience. Participants most commonly cited mo‘olelo (stories), oli (chants), mele (song), and cultural practices that have been passed on for generations as examples of the intergenerational knowledge that continues to persist as a means of ‘Āina connectedness. Through this innate connection came the deep responsibility to continue these practices as a form of health and resilience, while also demonstrating pride in one’s identity through a connection to these practices. Participants also described their inherited and acquired kuleana (birthright, privilege, responsibility) to perpetuate these practices for the betterment of their children and the health of our future generations.

When a person was in need of better health or healing, participants described the healing properties of ‘Āina and the importance of reconnecting with ‘Āina to foster health and healing. Participants from various communities and across the archipelago described the importance of ‘Āina as our lā‘au (medicine) and the way kūpuna (ancestors or elders of a grandparent’s generation) relied on ‘Āina for health and healing. Many participants described a strong desire to keep ‘Āina thriving to allow for the perpetuation of health and healing for their future generations. Similarly, a thriving ‘Āina was a demonstration of the resilience of a community, which also aligned with the philosophy that the health of the land is the health of people, a quote that was commonly cited during interviews.

### 3.5. Phase II: Development of the ‘Āina Connectedness Scale

Based on qualitative themes, consultation and collaboration with key members and leaders in Native Hawaiian communities, as well as a scoping review of the literature, our team designed the ‘Āina Connectedness Scale. In the first part of the scale, participants were asked to report the degree to which they feel connected to ‘Āina through behaviors, actions, and activities, with a specific focus on Native Hawaiian practices. This question was followed by participants reporting on the frequency in which participants would like to connect with ‘Āina through the forementioned behaviors, actions, and activities. Based on feedback provided during the cognitive interviews, these questions were reformatted to assess the way a person connects with ‘Āina through any of the listed behaviors, actions, activities, and Native Hawaiian practices, followed by an open-ended question that asked participants to prioritize ways they would like to connect with ‘Āina in the future.

The second part of the ‘Āina Connectedness Scale assessed barriers or variables that prevent a person from connecting with ‘Āina as well as variables or factors that encourage connecting with ‘Āina. To adhere to the original suggestion of using the same categorical response for all items, a Likert scale was utilized to determine the degree to which factors served as barriers or facilitators of connecting with ‘Āina. Based on feedback provided during the cognitive interviews and the complexity of these items, this portion of the survey was reformatted to include a checklist of items, with the first question assessing for barriers and inhibitors to connecting with ‘Āina, while the second question assessed for factors that encouraged connecting with ‘Āina. An open-ended question was also included to allow participants to provide a “short answer” of any additional barriers and facilitators of ‘Āina connectedness.

The last part of the ‘Āina Connectedness Scale assessed for ways of knowing, customs, beliefs, and traditions related to ‘Āina. This portion of the scale was the longest in length and was developed based on qualitative themes and adapted items from the Connectedness to Nature Scale [42], Awareness of Connectedness Scale [34], and the Hawaiian Culture Scale—Adolescent Version [33]. Cognitive interviews helped to explore the interpretation of each survey item, including the item structure, format, verbiage, and redundancy.

Components of the KOOKA Comprehensive Health Survey included demographics, the Nature Relatedness Scale (6-item) (NR-6) [43] to assess for construct validity, and health and health-related behaviors. To assess for health from a biomedical perspective, the comprehensive health survey included a section where participants were asked to indicate whether they have been previously diagnosed by a healthcare worker with a list of health conditions that were most cited by interviewees within the past year or ever in their lifetime. Other health and health-related behaviors included a range of measures, which included variables such as personal wellness; adversities of health, including land loss and historical trauma; measures of resilience; depressive measures; psychosocial factors; and measures such as food security.

## 4. Discussion

The findings from this study continue to emphasize the importance of maintaining ties and connections to one’s land. In general, qualitative findings continue to endorse the importance of connecting with nature or land as a mechanism for improving general health and wellbeing, with three major themes including: ‘Āina is everything; connection to ‘Āina is imperative to Native Hawaiian health; and intergenerational health, healing, and resilience of people and communities are reflected through intergenerational connectedness with ‘Āina. This worldview served as a foundation for this overall study, which aimed to report on the processes of developing the novel ‘Āina Connectedness Scale. ‘Āina connectedness, as a quantified measure, may play a critical role in health, thereby improving the function and longevity of land and health. This is timely given the continued concerns related to climate change and environmental changes.

These findings not only support the ongoing work of many Native Hawaiian scholars [1,2,4,7,8,9,10,11,12,13,14,15,44] but also extend to other Indigenous communities who have demonstrated the importance of land connectedness as a mechanism of cultural connectedness and as a way of life [32,34,35,36]. For instance, many other Indigenous worldviews take holistic approaches to health and emphasize the importance of maintaining respectful relationships and living in balance. This is showcased through concepts such as Hauora, as described by Māori, or the essence of the medicine wheel that have been used for generations by Indigenous Peoples of North America. Thus, the development of and endorsement of measures that assess land and nature connectedness are important from Native Hawaiian and Indigenous worldviews, with connection to land serving as a facilitator of wellbeing that may address socio-cultural determinants of health that stem from colonization and historical trauma.

When we acknowledge colonization, cultural and historical trauma, and systems of oppression as part of the adversities and core determinants of health that continue to influence the health and wellbeing of Native Hawaiians and Indigenous Peoples, we also acknowledge land connectedness and the continued survivance of Indigenous Peoples as part of the strengths and resiliency of Indigenous Peoples today [45,46,47,48]. This worldview also expands on the current operational definitions of resilience to highlight the importance of resistance, reclamation, reconnection, and resurgence of Indigenous epistemologies, ways of knowing, and ways of being. However, idealistically, these values and ways of being would not be implemented as a way of building resilience and overcoming adversity, but rather, a standard practice that acknowledges the right of every individual to simply be well. Thus, despite the fundamental human right to be well, Indigenous Peoples demonstrate their resilience through their survivance and continued connections to their ways of life.

The greatest strength of this study was the development of the ‘Āina Connectedness Scale with, for, and by Native Hawaiian communities. Despite the strengths of this study, limitations must also be acknowledged. While the research team attempted to include a diverse sample of Native Hawaiian community members and leaders throughout the archipelago of Hawai‘i, the findings may be limited to those who participated in this study and may not be generalizable to other Native Hawaiian members and communities. To account for this limitation, the research team recruited a sample of 40 Native Hawaiian adults to ensure theoretical saturation.

The time period in which the interviews were conducted and the comprehensive survey was developed may have potential impacts for the long-term understanding of ‘Āina connectedness. Not only are these findings limited to cross-section data, but they may also be impacted by other contextual factors such as the heightened awareness of Native Hawaiian movements, such as the protection of Mauna a Wakea, as well as impacts resulting from the COVID-19 pandemic. Approximately three-fourths of qualitative interviews and focus groups were conducted prior to the significant impacts of the COVID-19 global pandemic in Hawai‘i. On the other hand, 10 of the interviews were conducted during the COVID-19 global pandemic. To ensure the safety and protection of participants, all interviews during the COVID-19 pandemic took place via Zoom, which may have impacted the general flow and structure of the interview. Despite the research team identifying theoretical saturation related to ‘Āina and ‘Āina connectedness, the COVID-19 pandemic also created nuances that complicated the ability of interviewees to connect with ‘Āina through certain practices. COVID-19 also brought to light many pressing inequities that were heightened during the pandemic, which may have informed measures that were included in the KOOKA comprehensive health survey.

In addition to these limitations, the research team identified lessons learned throughout the research process and compiled a list of questions that we responded to as a research team throughout the research process (See Appendix A). Some of these lessons learned are further described. First, a community-prioritized—and thus, a community-based—research approach that is culturally grounded and takes a decolonized approach to research takes great time and effort to truly build rapport and relationships to ensure the research process is driven with, for, and by communities. As such, Indigenous epistemologies take precedence over other research agendas and require transparency about data governance and the expected time to undergo research processes, including financial logistics. This may shed light on some of the incongruences that may exist between research at the academy and research in communities.

## 5. Conclusions

The Ke Ola O Ka ‘Āina: ‘Āina Connectedness Scale as a quantified measure will address current gaps in the literature and in the field of health equity research for Native Hawaiian health. This work and the development of the ‘Āina Connectedness Scale validates the importance of intergenerational/ancestral knowledge, values, and ways of knowing that honor a deep relationship with nature and land and has implications for communities (at large) who have experienced significant disconnections to land. Stronger relationships to the land will help to inform efforts that aim to heal socio-cultural determinants of health through connections with land. This study has implications for Indigenous communities who have experienced significant disconnections to land as a result of colonization and historical trauma. In particular, stronger relationships with/to the land will help to inform efforts that aim to heal trauma through (re)connections with land.

## 6. Patents and Copyright

Ke Ola O Ka ‘Āina: ‘Āina Connectedness Scale belongs to the Ke Ola O Ka ‘Āina Research Team and Thought Partners.

## Figures and Tables

**Table 1:** Participant Characteristics.

Characteristics	Values *n* (%)
Gender	
Kāne or Male	20 (50%)
Wāhine or Female	20 (50%)
Ages	
Mākua (Parent or of a parent’s generation)	13 (33%)
Kūpuna (Grandparent or of a grandparent’s generation)	27 (67%)
One Hānau (Birthplace, homeland)	
Hawai‘i Island	7 (18%)
Lāna‘i and Moloka‘i	2 (5%)
Maui Island	8 (20%)
O‘ahu Island	17 (43%)
Kaua‘i Island	6 (15%)

**Table 2:** Summary of Themes.

Theme	Codebook Definition	Example Quotes
(1) ‘Āina is everything	(1) ‘Āina loosely translates to land and that which feeds. (2) ‘Āina is everything including us as people. (3) ‘Āina extends to include anything that nourishes us physically, mentally, spiritually, and emotionally.	“That which feeds. But I think of ‘āina as not only land but the interconnectedness of all things… we are, we as humans, derive from the ‘Āina” (Kaua‘i Interviewee) “I am the ‘āina”. (Hawai‘i Island Interviewee) “‘Āina is everything. And is anything that nourishes us. ‘Āina is the land-from the mountains, to the sea, to us as people. It’s all connected…so when we connect with ‘Āina, we connect to each other”. (O‘ahu Interviewee)
(2) Connection to ‘Āina is imperative to Native Hawaiian health	Connection to ‘Āina is imperative to health due to the values and ways of life/knowing that are inextricably linked to concepts such as: (1) Genealogy (2) Respect and kuleana (3) Connection to each other, our ‘ohana, communities, and lāhui	“It’s like that story about Hāloanakalaukapalili, who is our older sibling. The elder sibling to kānaka…I might not know my great-great-great- great-great grandfather, but I still know I am related to ‘Āina because I am Kanaka Maoli”. (O‘ahu Interviewee) “We have a kuleana to ‘Āina and to Kānaka”. (Maui Interviewee) “When we connect with ‘Aina, we connect with land and when we connect with land, we connect with each other”. (Hawai‘i Island Interviewee)
(3) Intergenerational health, healing, and resilience of people and communities are reflected through intergenerational connectedness with ‘Āina	(1) ‘Āina connectedness is the result of intergenerational knowledge and stewardship. (2) We must care for the land to allow for health and healing for our future generations. (3) Thriving ‘Āina is a demonstration of the health and resilience of people.	“Mālama (take care of) the kaona (hidden and deeper meanings) from kūpuna (ancestors, or of a grandparents generation) as we are beneficiaries of that kaona”. (Lāna‘i/Molokai Interviewee) “Health is how healthy we keep our ‘Āina. It needs to be pono…It’s our inheritance to mālama and make our next generation proud to be Hawaiian” (Maui Interviewee) “So long as we mālama ‘Āina, ‘Āina will thrive. And just like ‘Āina, we too, will thrive”. (O‘ahu Interviewee)

## Data Availability

The stories and data that are shared in this research study belong to the various communities, organizations, and individuals who are represented in this paper.

## Appendix A. Questions We Have Responded to during the KOOKA Research Process

We have the Waimānalo Pono Research Hui rules of engagement with research. Are you willing to undergo this process?

What is the budget? How was the budget created? How will the budget be shared?

What are the benefits and incentives for participants?

Who is leading the survey? Why was the survey first created?

Who wrote the survey?

How long is the survey? How were the survey questions selected?

How will the data be used? Who will have access to data?

What are the plans for collecting data moving forward?

What programs will come from the survey? What policies will come from the survey?

What university researchers are involved? Who are your community partners?

Where are partners during meetings/zoom calls/presentations?

Who specifically benefited from this research?

How did other communities benefit?

Who has published this work?

How will the team obtain permission to use data? (Now and in the future)
